# Importance of Religious Understanding in Psychiatric Evaluation: A Case Report of a Palestinian Christian Patient

**DOI:** 10.1192/j.eurpsy.2025.1293

**Published:** 2025-08-26

**Authors:** N. Marzouqa, N. AlShaikh

**Affiliations:** 1 Bethlehem Psychiatric Hospital, Bethlehem, Palestinian, State of

## Abstract

**Introduction:**

Religion plays a big role in daily life in Palestine: it helps give meaning and purpose and creates communities and mutual understanding. Thus, individuals frequently bring up various thoughts and ideas with religious themes and undertones during psychiatric interviews, reflecting their inner and outer experiences. Religious references can be misunderstood when discussed by individuals of religious minorities, such as Palestinian Christians, who comprise around 1% of the Palestinian population in the West Bank (2023 Report on International Religious Freedom). Studies on the mental health of Palestinian minorities are almost nonexistent.

**Objectives:**

Examining the responsibility of psychiatrists to provide culturally sensitive care to their patients, including religious minorities.

**Methods:**

A case report of a Palestinian Christian patient admitted to an acute psychiatric ward in Palestine. This 37-year-old male patient, who has struggled with mental health issues for the past 4 years, presented to Bethlehem Psychiatric Hospital after 10 days of suspiciousness, disturbed behavior, and verbal aggression toward family members. On examination, the patient was found to have a tense elevated mood with persecutory, reference, and grandiose delusions. The patient frequently brought content and references from the liturgy and the New Testament. Blood and urine tests were within normal range and showed no substance use. The patient was started on Olanzapine 15mg and Valproate 400mg.

**Results:**

A review of his hospital file revealed that after 2 weeks on medications, he no longer displayed ideas of delusional intensity. However, the patient mentioned events and lessons from the books of Revelation and Acts of the Apostles of the Christian Bible. He had a particular fascination with their relation to the Old Testament and the image of “tongues of fire” from the Pentecost. Due to his prior psychotic symptoms and limited overall knowledge of Christianity, it was difficult for many staff members to assess his thought content. These challenges were overcome by discussions with Christian hospital staff, gathering collaterals from his family, and self-education. The patient was using moments of transition in the Bible to describe his transition to a non-psychotic state. He was discharged soon after and referred to a community mental health center.

**Conclusions:**

It is common for Arab patients to have psychiatric pathology of religious themes. These are important to distinguish from cultural expressions of emotions and experiences. To provide inclusive and comprehensive care, it is essential for clinicians to have an appropriate understanding of the religions, spirituality, and diversity of the populations they are serving.

**Disclosure of Interest:**

None Declared

